# Supplementary material for: Analyzing Precision Medicine Utilization with Real-World Data: A Scoping Review
Source: J Pers Med. 2022 Apr 1;12(4):557. doi: 10.3390/jpm12040557 (PMC9025578; doi:10.3390/jpm12040557)
Supplement: Supplementary file 1 [file jpm-12-00557-s001.zip › jpm-1625509-supplementary/jpm-1625509 - Supplementary Materials - Proofreading.pdf]

Journal of *Personalized Medicine*

MDPI

Supplementary Materials

S1. Search Terms

(("Whole Genome Sequencing"[Mesh]) OR "Whole Exome Sequencing"[Mesh] OR "tumor sequencing"[All Fields] OR "genetic test*"[All Fields] OR "genomic test*"[All Fields] OR "pharmacogenetic test*"[All Fields] OR "pharmacogenomic test*"[All Fields] OR "Gene Expression Profiling"[All Fields] OR "Sequence Analysis"[MeSH Terms]) AND ("utilization"[All Fields] OR "Health Services Research"[MeSH Terms] OR "genetic testing/economics"[MeSH Terms] OR "precision medicine/economics"[MeSH Terms] OR "gene expression profiling/economics"[MeSH Terms] OR "medicare/economics"[MeSH Terms] OR "genetic testing/trends"[MeSH Terms] OR "precision medicine/trends"[MeSH Terms] OR "gene expression profiling/trends"[MeSH Terms] OR "medicare/trends"[MeSH Terms] OR "testing trends"[All Fields] OR "claims data"[All Fields] OR "Medicare claims"[All Fields]) AND ("real-world data"[All Fields] OR "real-world evidence"[All Fields] OR "Big Data"[All Fields] OR "Medical Records"[All Fields] OR "Databases as Topic"[MeSH Terms] OR "Optum"[All Fields] OR "Truven"[All Fields] OR "Flatiron"[All Fields] OR "SEER Program"[MeSH Terms] OR "genetic testing/statistics and numerical data"[MeSH Terms] OR "precision medicine/statistics and numerical data"[MeSH Terms] OR "gene expression profiling/statistics and numerical data"[MeSH Terms] OR "medicare/statistics and numerical data"[MeSH Terms] OR "Medicare claims"[All Fields] OR "data set"[All Fields] OR "claims data"[All Fields] OR "electronic health record*"[All Fields] OR "electronic medical record*"[All Fields]) AND (2015/1/1:3000/12/12[pdat]).

Table S2. Data Abstracted

Data variables abstracted and coding are presented in Table S2 Excel spreadsheet.

S3. Studies Included

11. Roberts, M.C.; Kurian, A.W.; Petkov, V.I. Uptake of the 21-Gene Assay Among Women With Node-Positive, Hormone Receptor-Positive Breast Cancer. *J. Natl. Compr. Cancer Netw.* **2019**, *17*, 662–668, doi:10.6004/jnccn.2018.7266.
17. Abul-Husn, N.S.; Manickam, K.; Jones, L.K.; Wright, E.A.; Hartzel, D.N.; Gonzaga-Jauregui, C.; O'Dushlaine, C.; Leader, J.B.; Kirchner, H.L.; Lindbuchler, D.M.; et al. Genetic identification of familial hypercholesterolemia within a single U.S. health care system. *Sci.* **2016**, *354*, aaf7000, doi:10.1126/science.aaf7000.
18. Altman, A.M.; Marmor, S.; Tuttle, T.M.; Hui, J.Y.C. 21-Gene Recurrence Score Testing in HER2-positive Patients. *Clin. Breast Cancer* **2019**, *19*, 126–130, doi:10.1016/j.clbc.2018.11.011.
19. Anderson, H.D.; Crooks, K.R.; Kao, D.P.; Aquilante, C.L. The landscape of pharmacogenetic testing in a US managed care population. *Genet. Med.* **2020**, *22*, 1247–1253, doi:10.1038/s41436-020-0788-3.
20. Armstrong, J.; Toscano, M.; Kotchko, N.; Friedman, S.; Schwartz, M.D.; Virgo, K.S.; Lynch, K.; Andrews, J.E.; Loi, C.X.A.; Bauer, J.E.; et al. Utilization and Outcomes of BRCAGenetic Testing and Counseling in a National Commercially Insured Population. *JAMA Oncol.* **2015**, *1*, 1251–1260, doi:10.1001/jamaoncol.2015.3048.
21. Bardakjian, T.M.; Helbig, I.; Quinn, C.; Elman, L.B.; McCluskey, L.F.; Scherer, S.S.; Gonzalez-Alegre, P. Genetic test utilization and diagnostic yield in adult patients with neurological disorders. *neurogenetics* **2018**, *19*, 105–110, doi:10.1007/s10048-018-0544-x.
22. Benitez, J.; Cool, C.L.; Scotti, D.J. Use of combinatorial pharmacogenomic guidance in treating psychiatric disorders. *Pers. Med.* **2018**, *15*, 481–494, doi:10.2217/pme-2018-0074.
23. Bhutiani, N.; Vuong, B.; Egger, M.E.; Eldredge-Hindy, H.; McMasters, K.M.; Ajkay, N. Evaluating patterns of utilization of gene signature panels and impact on treatment patterns in patients with ductal carcinoma in situ of the breast. *Surg.* **2019**, *166*, 509–514, doi:10.1016/j.surg.2019.04.044.
24. Blaes, A.H.; I Jewett, P.; McKay, K.; Riley, D.; Jatoi, I.; Trentham-Dietz, A.; Chrischilles, E.; Klemp, J.R. Factors associated with genetic testing in a cohort of breast cancer survivors. *Breast J.* **2019**, *25*, 1241–1244, doi:10.1111/tbj.13440.
25. Blagec, K., Kuch, W., Samwald, M. The Importance of Gene-Drug-Drug-Interactions in Pharmacogenomics Decision Support: An Analysis Based on Austrian Claims Data. In *Health Informatics Meets eHealth*; Schreier, G., Ammenwerth, E., Hörbst, A., Hayn, D., Eds.; Studies in Health Technology and Informatics; IOS Press: Amsterdam, The Netherlands, 2017; 236, 121-7. Available online: https://ebooks.iospress.nl/publication/46468 (accessed on 18 February 2022).
26. Byfield, S.D.; Wei, H.; DuCharme, M.; Lancaster, J.M. Economic impact of multigene panel testing for hereditary breast and ovarian cancer. *J. Comp. Eff. Res.* **2021**, *10*, 207–217, doi:10.2217/cer-2020-0192.

27. Caplan, E.O.; Wong, W.B.; Ferries, E.; Hulinsky, R.; Brown, V.T.; Bordenave, K.; Suehs, B.T. Novel Approach Using Administrative Claims to Evaluate Trends in Oncology Multigene Panel Testing for Patients Enrolled in Medicare Advantage Health Plans. *JCO Precis. Oncol.* **2021**, *5*, 792–801, doi:10.1200/po.20.00422.
28. Chen, Z.; Kolor, K.; Grosse, S.D.; Rodriguez, J.L.; Lynch, J.A.; Green, R.F.; Dotson, W.D.; Bowen, M.S.; Khoury, M.J. Trends in utilization and costs of BRCA testing among women aged 18–64 years in the United States, 2003–2014. *Genet. Med.* **2018**, *20*, 428–434, doi:10.1038/gim.2017.118.
29. Childers, K.K.; Maggard-Gibbons, M.; Macinko, J.; Childers, C.P. National Distribution of Cancer Genetic Testing in the United States. *JAMA Oncol.* **2018**, *4*, 876, doi:10.1001/jamaoncol.2018.0340.
30. Chitty, L.S.; Wright, D.; Hill, M.; I Verhoef, T.; Daley, R.; Lewis, C.; Mason, S.; McKay, F.; Jenkins, L.; Howarth, A.; et al. Uptake, outcomes, and costs of implementing non-invasive prenatal testing for Down's syndrome into NHS maternity care: prospective cohort study in eight diverse maternity units. *BMJ* **2016**, *354*, i3426, doi:10.1136/bmj.i3426.
31. Cress, R.D.; Chen, Y.S.; Morris, C.R.; Chew, H.; Kizer, K.W. Underutilization of gene expression profiling for early-stage breast cancer in California. *Cancer Causes Control* **2016**, *27*, 721–727, doi:10.1007/s10552-016-0743-4.
32. Dalal, A.A.; Guerin, A.; Mutebi, A.; Culver, K.W. Economic analysis of BRAF gene mutation testing in real world practice using claims data: costs of single gene versus panel tests in patients with lung cancer. *J. Med Econ.* **2018**, *21*, 649–655, doi:10.1080/13696998.2018.1450261.
33. DeFrancesco, M.S.; Waldman, R.N.; Pearlstone, M.M.; Karanik, D.; Bernhisel, R.; Logan, J.; Alico, L.; Adkins, R.T. Hereditary Cancer Risk Assessment and Genetic Testing in the Community-Practice Setting. *Obstet. Gynecol.* **2018**, *132*, 1121–1129, doi:10.1097/aog.0000000000002916.
34. Desai, S.; Jena, A.B. Do celebrity endorsements matter? Observational study of BRCA gene testing and mastectomy rates after Angelina Jolie's New York Times editorial. *BMJ* **2016**, *355*, i6357, doi:10.1136/bmj.i6357.
35. Dinan, M.A.; Mi, X.; Reed, S.D.; Hirsch, B.R.; Lyman, G.H.; Curtis, L.H. Initial Trends in the Use of the 21-Gene Recurrence Score Assay for Patients With Breast Cancer in the Medicare Population, 2005-2009. *JAMA Oncol.* **2015**, *1*, 158–66, doi:10.1001/jamaoncol.2015.43.
36. Dinan, M.A.; Mi, X.; Reed, S.D.; Lyman, G.H.; Curtis, L.H. Association Between Use of the 21-Gene Recurrence Score Assay and Receipt of Chemotherapy Among Medicare Beneficiaries With Early-Stage Breast Cancer, 2005-2009. *JAMA Oncol.* **2015**, *1*, 1098–1109, doi:10.1001/jamaoncol.2015.2722.
37. Dinan, M.A.; Wilson, L.; Reed, S.D. Chemotherapy Costs and 21-Gene Recurrence Score Genomic Testing Among Medicare Beneficiaries With Early-Stage Breast Cancer, 2005 to 2011. *J. Natl. Compr. Cancer Netw.* **2019**, *17*, 245–254, doi:10.6004/jnccn.2018.7097.
38. Dinan, M.A.; Wilson, L.E.; Reed, S.D.; Griggs, J.J.; Norton, E.C. Association of 21-Gene Assay (OncotypeDX) Testing and Receipt of Chemotherapy in the Medicare Breast Cancer Patient Population Following Initial Adoption. *Clin. Breast Cancer* **2020**, *20*, 487–494.e1, doi:10.1016/j.clbc.2020.05.010.
39. Dubrovsky, E.; Raymond, S.; Chun, J.; Fong, A.; Patel, N.; Guth, A.; Schnabel, F. Genomic testing in early stage invasive male breast cancer: An NCDB analysis from 2008 to 2014. *Breast J.* **2019**, *25*, 425–433, doi:10.1111/tbj.13235.
40. El Rouby, N.; Alrwisan, A.; Langaee, T.; Lipori, G.; Angiolillo, D.J.; Franchi, F.; Riva, A.; Elsey, A.; Johnson, J.A.; Cavallari, L.H.; et al. Clinical Utility of Pharmacogene Panel-Based Testing in Patients Undergoing Percutaneous Coronary Intervention. *Clin. Transl. Sci.* **2019**, *13*, 473–481, doi:10.1111/cts.12729.
41. Fohner, A.E.; Ranatunga, D.K.; Thai, K.K.; Lawson, B.L.; Risch, N.; Oni-Orisan, A.; Jelalian, A.T.; Rettie, A.E.; Liu, V.X.; Schaefer, C.A. Assessing the clinical impact of CYP2C9 pharmacogenetic variation on phenytoin prescribing practice and patient response in an integrated health system. *Pharmacogenetics Genom.* **2019**, *29*, 192–199, doi:10.1097/fpc.0000000000000383.
42. Franc, B.L.; Copeland, T.P.; Thombley, R.; Park, M.; Marafino, B.; Dean, M.; Boscardin, W.J.; Rugo, H.S.; Dudley, R.A. Geographic and Patient Characteristics Associated With Election of Prophylactic Mastectomy in Young Breast Cancer Patients With Early Disease. *Am. J. Clin. Oncol.* **2018**, *41*, 1037–1042, doi:10.1097/coc.0000000000000446.
43. Geddes, G.C.; Basel, D.; Frommelt, P.; Kinney, A.; Earing, M. Genetic Testing Protocol Reduces Costs and Increases Rate of Genetic Diagnosis in Infants with Congenital Heart Disease. *Pediatr. Cardiol.* **2017**, *38*, 1465–1470, doi:10.1007/s00246-017-1685-7.
44. Guo, F.; Bs, M.S.; Fuchs, E.L.; Berenson, A.B.; Kuo, Y. BRCA testing in unaffected young women in the United States, 2006-2017. *Cancer* **2020**, *126*, 337–343, doi:10.1002/cncr.32536.
45. Guo, F.; Scholl, M.; Fuchs, E.L.; Wong, R.; Kuo, Y.-F.; Berenson, A.B. Trends in Positive BRCA Test Results Among Older Women in the United States, 2008-2018. *JAMA Netw. Open* **2020**, *3*, e2024358, doi:10.1001/jamanetworkopen.2020.24358.
46. Hefti, E.; Jacobs, D.M.; Rana, K.; Blanco, J.G. Analysis of outpatient HER2 testing in New York state using the statewide planning and research cooperative system. *Pharmacogenomics* **2018**, *19*, 1395–1401, doi:10.2217/pgs-2018-0120.
47. Huang, M.; Kamath, P.; Schlumbrecht, M.; Miao, F.; Driscoll, D.; Oldak, S.; Slomovitz, B.; Koru-Sengul, T.; George, S. Identifying disparities in germline and somatic testing for ovarian cancer. *Gynecol. Oncol.* **2019**, *153*, 297–303, doi:10.1016/j.ygyno.2019.03.007.
48. Hui, L.; Hutchinson, B.; Poulton, A.; Halliday, J. Population-based impact of noninvasive prenatal screening on screening and diagnostic testing for fetal aneuploidy. *Genet. Med.* **2017**, *19*, 1338–1345, doi:10.1038/gim.2017.55.

49. Hull, L.E.; Lynch, J.A.; Berse, B.B.; DuVall, S.L.; Chun, D.S.; Venne, V.L.; Efimova, O.V.; Icardi, M.S.; Kelley, M.J. Clinical Impact of 21-Gene Recurrence Score Test Within the Veterans Health Administration: Utilization and Receipt of Guideline-Concordant Care. *Clin. Breast Cancer* **2017**, *18*, 135–143, doi:10.1016/j.clbc.2017.11.018.
50. Katz, S.J.; Bondarenko, I.; Ward, K.C.; Hamilton, A.S.; Morrow, M.; Kurian, A.W.; Hofer, T.P. Association of Attending Surgeon With Variation in the Receipt of Genetic Testing After Diagnosis of Breast Cancer. *JAMA Surg.* **2018**, *153*, 909, doi:10.1001/jamasurg.2018.2001.
51. Nzale, S.K.; Weeks, W.B.; Ouafik, L.; Rouquette, I.; Beau-Faller, M.; Lemoine, A.; Bringuier, P.-P.; Soriano, A.-G.L.C.; Barlesi, F.; Ventelou, B. Inequity in access to personalized medicine in France: Evidences from analysis of geo variations in the access to molecular profiling among advanced non-small-cell lung cancer patients: Results from the IFCT Biomarkers France Study. *PLoS ONE* **2020**, *15*, e0234387, doi:10.1371/journal.pone.0234387.
52. Kolor, K.; Chen, Z.; Grosse, S.D.; Rodriguez, J.L.; Green, R.F.; Dotson, W.D.; Bowen, M.S.; Lynch, J.A.; Khoury, M.J.; BRCA Genetic Testing and Receipt of Preventive Interventions among Women Aged 18–64 Years with Employer-Sponsored Health Insurance in Nonmetropolitan and Metropolitan Areas - United States, 2009–2014, 2017. Centers for Diseases Control and Prevention Website. Available online: https://www.cdc.gov/mmwr/volumes/66/ss/ss6615a1.htm (accessed on 18 February 2022).
53. Larson, K.L.; Huang, B.; Chen, Q.; Tucker, T.; Schuh, M.; Arnold, S.M.; Kolesar, J.M. EGFR testing and erlotinib use in non-small cell lung cancer patients in Kentucky. *PLoS ONE* **2020**, *15*, e0237790, doi:10.1371/journal.pone.0237790.
54. Li, H.; Fan, J.; Vitali, F.; Berghout, J.; Aberasturi, D.; Li, J.; Wilson, L.; Chiu, W.; Pumarejo, M.; Han, J.; et al. Novel disease syndromes unveiled by integrative multiscale network analysis of diseases sharing molecular effectors and comorbidities. *BMC Med Genom.* **2018**, *11*, 112, doi:10.1186/s12920-018-0428-9.
55. Liede, A.; Cai, M.; Crouter, T.F.; Niepel, D.; Callaghan, F.; Evans, D.G. Risk-reducing mastectomy rates in the US: a closer examination of the Angelina Jolie effect. *Breast Cancer Res. Treat.* **2018**, *171*, 435–442, doi:10.1007/s10549-018-4824-9.
56. Lynch, J.A.; Berse, B.; Coomer, N.; Kautter, J. 21-Gene recurrence score testing among Medicare beneficiaries with breast cancer in 2010–2013. *Genet. Med.* **2017**, *19*, 1134–1143, doi:10.1038/gim.2017.19.
57. Lynch, J.A.; Berse, B.; Dotson, W.D.; Khoury, M.J.; Coomer, N.; Kautter, J. Utilization of genetic tests: analysis of gene-specific billing in Medicare claims data. *Genet. Med.* **2017**, *19*, 890–899, doi:10.1038/gim.2016.209.
58. Mackenzie, S.J.; Lin, C.C.; Todd, P.K.; Burke, J.F.; Callaghan, B.C. Genetic testing utilization for patients with neurologic disease and the limitations of claims data. *Neurol. Genet.* **2020**, *6*, e405, doi:10.1212/nxg.0000000000000405.
59. Mathias, P.C.; Hendrix, N.; Wang, W.-J.; Keyloun, K.; Khelifi, M.; Tarczy-Hornoch, P.; Devine, B. Characterizing Pharmacogenomic-Guided Medication Use With a Clinical Data Repository. *Clin. Pharmacol. Ther.* **2017**, *102*, 340–348, doi:10.1002/cpt.611.
60. McCuaig, J.M.; Care, M.; Ferguson, S.E.; Kim, R.H.; Stockley, T.L.; Metcalfe, K.A. Year 1: Experiences of a tertiary cancer centre following implementation of reflex BRCA1 and BRCA2 tumor testing for all high-grade serous ovarian cancers in a universal healthcare system. *Gynecol. Oncol.* **2020**, *158*, 747–753, doi:10.1016/j.ygyno.2020.06.507.
61. Mittmann, N.; Earle, C.C.; Cheng, S.Y.; Julian, J.A.; Rahman, F.; Seung, S.J.; Levine, M.N. Population-Based Study to Determine the Health System Costs of Using the 21-Gene Assay. *J. Clin. Oncol.* **2018**, *36*, 238–243, doi:10.1200/jco.2017.74.2577.
62. Muller, C.; Lee, S.M.; Barge, W.; Siddique, S.M.; Berera, S.; Wideroff, G.; Tondon, R.; Chang, J.; Peterson, M.; StollJ.; et al. Low Referral Rate for Genetic Testing in Racially and Ethnically Diverse Patients Despite Universal Colorectal Cancer Screening. *Clin. Gastroenterol. Hepatol.* **2018**, *16*, 1911–1918.e2, doi:10.1016/j.cgh.2018.08.038.
63. O'Neill, S.C.; Isaacs, C.; Chao, C.; Tsai, H.-T.; Liu, C.; Ekezue, B.F.; Selvam, N.; Kessler, L.G.; Schwartz, M.D.; Lobo, T.; et al. Adoption of Gene Expression Profiling for Breast Cancer in US Oncology Practice for Women Younger Than 65 Years. *J. Natl. Compr. Cancer Netw.* **2015**, *13*, 1216–1224, doi:10.6004/jnccn.2015.0150.
64. O'neill, S.C.; Isaacs, C.; Lynce, F.; Graham, D.M.A.; Chao, C.; Sheppard, V.B.; Zhou, Y.; Liu, C.; Selvam, N.; Schwartz, M.D.; et al. Endocrine therapy initiation, discontinuation and adherence and breast imaging among 21-gene recurrence score assay-eligible women under age 65. *Breast Cancer Res.* **2017**, *19*, 45, doi:10.1186/s13058-017-0837-2.
65. Orucevic, A.; Heidel, R.E.; Bell, J.L. Utilization and impact of 21-gene recurrence score assay for breast cancer in clinical practice across the United States: lessons learned from the 2010 to 2012 National Cancer Data Base analysis. *Breast Cancer Res. Treat.* **2016**, *157*, 427–435, doi:10.1007/s10549-016-3833-9.
66. Pace, L.E.; Baum, C.F.; Horvath, K.; Raja, S.; Cohen, J.; Hawkins, S.S. BRCA1/2 Testing in Massachusetts Among Women With Private Insurance or Medicaid, 2011–2015. *Med Care* **2020**, *58*, 963–967, doi:10.1097/mlr.0000000000001405.
67. Palazzo, L.L.; Sheehan, D.F.; Tramontano, A.C.; Kong, C.Y. Disparities and Trends in Genetic Testing and Erlotinib Treatment among Metastatic Non–Small Cell Lung Cancer Patients. *Cancer Epidemiology Biomarkers Prev.* **2019**, *28*, 926–934, doi:10.1158/1055-9965.epi-18-0917.
68. Parkhurst, E.; Calonico, E.; Abboy, S. Utilization of Genetic Testing for RET Mutations in Patients with Medullary Thyroid Carcinoma: a Single-Center Experience. *J. Genet. Couns.* **2018**, *27*, 1411–1416, doi:10.1007/s10897-018-0273-1.
69. Pavey, A.R.; Bodian, D.L.; Vilboux, T.; Khromykh, A.; Hauser, N.S.; Huddleston, K.; Klein, E.; Black, A.; Kane, M.S.; Iyer, R.K.; et al. Utilization of genomic sequencing for population screening of immunodeficiencies in the newborn. *Genet. Med.* **2017**, *19*, 1367–1375, doi:10.1038/gim.2017.57.
70. Perlis, R.H.; Mehta, R.; Edwards, A.M.; Tiwari, A.; Imbens, G.W. Pharmacogenetic testing among patients with mood and anxiety disorders is associated with decreased utilization and cost: A propensity-score matched study. *Depression Anxiety* **2018**, *35*, 946–952, doi:10.1002/da.22742.

71. Petelin, L.; James, P.A.; Trainer, A.H. Changing landscape of hereditary breast and ovarian cancer germline genetic testing in Australia. *Intern. Med. J.* **2018**, *48*, 1269–1272, doi:10.1111/imj.14058.
72. Pokharel, H.P.; Hacker, N.F.; Andrews, L. Changing patterns of referrals and outcomes of genetic participation in gynaecological-oncology multidisciplinary care. *Aust. New Zealand J. Obstet. Gynaecol.* **2016**, *56*, 633–638, doi:10.1111/ajo.12504.
73. Potosky, A.L.; O'Neill, S.C.; Isaacs, C.; Tsai, H.-T.; Chao, C.; Liu, C.; Ekezue, B.F.; Selvam, N.; Kessler, L.G.; Zhou, Y.; et al. Population-based study of the effect of gene expression profiling on adjuvant chemotherapy use in breast cancer patients under the age of 65 years. *Cancer* **2015**, *121*, 4062–4070, doi:10.1002/cncr.29621.
74. Poulton, A.; Lewis, S.; Hui, L.; Halliday, J.L. Prenatal and preimplantation genetic diagnosis for single gene disorders: A population-based study from 1977 to 2016. *Prenat. Diagn.* **2018**, *38*, 904–910, doi:10.1002/pd.5352.
75. Ray, G.T.; Mandelblatt, J.; A Habel, L.; Ramsey, S.; Kushi, L.H.; Li, Y.; A Lieu, T. Breast cancer multigene testing trends and impact on chemotherapy use. *Am. J. Manag. Care* **2016**, *22*, e153–60.
76. Ritter, A.; Bedoukian, E.; Berger, J.H.; Copenheaver, D.; Gray, C.; Krantz, I.; Izumi, K.; Juusola, J.; Leonard, J.; Lin, K.; et al. Clinical utility of exome sequencing in infantile heart failure. *Genet. Med.* **2019**, *22*, 423–426, doi:10.1038/s41436-019-0654-3.
77. Roberts, M.C.; Dusetzina, S.B. Use and Costs for Tumor Gene Expression Profiling Panels in the Management of Breast Cancer From 2006 to 2012: Implications for Genomic Test Adoption Among Private Payers. *J. Oncol. Pr.* **2015**, *11*, 273–277, doi:10.1200/jop.2015.003624.
78. Roberts, M.C.; Dusetzina, S. The effect of a celebrity health disclosure on demand for health care: trends in BRCA testing and subsequent health services use. *J. Community Genet.* **2017**, *8*, 141–146, doi:10.1007/s12687-017-0295-7.
79. Roberts, M.C.; Weinberger, M.; Dusetzina, S.; Dinan, M.A.; Reeder-Hayes, K.E.; Carey, L.A.; Troester, M.A.; Wheeler, S.B. Racial Variation in the Uptake of Oncotype DX Testing for Early-Stage Breast Cancer. *J. Clin. Oncol.* **2016**, *34*, 130–138, doi:10.1200/jco.2015.63.2489.
80. Schoen, C.; Santolaya-Forgas, J.; Genc, M.; Ashkinadze, E. Differential utilization of expanded genetic screening tests in patients of reproductive ages from private and academic practices. *J. Périnat. Med.* **2015**, *43*, 689–93, doi:10.1515/jpm-2014-0160.
81. Shaikh, T.; Handorf, E.A.; Meyer, J.E.; Hall, M.J.; Esnaola, N.F. Mismatch Repair Deficiency Testing in Patients With Colorectal Cancer and Nonadherence to Testing Guidelines in Young Adults. *JAMA Oncol.* **2018**, *4*, e173580, doi:10.1001/jamaoncol.2017.3580.
82. Singer, J.; Hanna, J.W.; Visaria, J.; Gu, T.; McCoy, M.; Kloos, R.T. Impact of a gene expression classifier on the long-term management of patients with cytologically indeterminate thyroid nodules. *Current medical research and opinion 32*, 1225–32, doi:10.6084/m9.figshare.3160507.
83. Sivapiragasam, A.; Kumar, P.A.; Sokol, E.S.; Albacker, L.A.; Killian, J.K.; Ramkissoon, S.H.; Huang, R.S.P.; Severson, E.A.; Brown, C.A.; Danziger, N.; et al. Predictive Biomarkers for Immune Checkpoint Inhibitors in Metastatic Breast Cancer. *Cancer Med.* **2021**, *10*, 53–61, doi:10.1002/cam4.3550.
84. Stein, Q.P.; for the Inborn Errors of Metabolism Collaborative; Vockley, C.W.; Edick, M.J.; Zhai, S.; Hiner, S.J.; Loman, R.S.; Davis-Keppen, L.; Zuck, T.A.; Cameron, C.A.; et al. An Exploration of Genetic Test Utilization, Genetic Counseling, and Consanguinity within the Inborn Errors of Metabolism Collaborative (IBEMC). *J. Genet. Couns.* **2017**, *26*, 1238–1243, doi:10.1007/s10897-017-0100-0.
85. Tanner, J.-A.; Brown, L.C.; Yu, K.; Li, J.; Dechairo, B.M. Canadian Medication Cost Savings Associated with Combinatorial Pharmacogenomic Guidance for Psychiatric Medications. *Clin. Outcomes Res.* **2019**, *ume 11*, 779–787, doi:10.2147/ceor.s224277.
86. Tiller, G.E.; Kershberg, H.B.; Goff, J.; Coffeen, C.; Liao, W.; Sehnert, A.J. Women's views and the impact of noninvasive prenatal testing on procedures in a managed care setting. *Prenat. Diagn.* **2015**, *35*, 428–433, doi:10.1002/pd.4495.
87. Vaeth, S.; Christensen, R.; Dunø, M.; Lildballe, D.L.; Thorsen, K.; Vissing, J.; Svenstrup, K.; Hertz, J.M.; Andersen, H.; Jensen, U.B. Genetic analysis of Charcot-Marie-Tooth disease in Denmark and the implementation of a next generation sequencing platform. *Eur. J. Med Genet.* **2018**, *62*, 1–8, doi:10.1016/j.ejmg.2018.04.003.

Table S4. Preferred Reporting Items for Systematic reviews and Meta-Analyses extension for Scoping Reviews (PRISMA-ScR) Checklist.

Section	Item	PRISMA-ScR Checklist Item	Reported on Page #
		TITLE	
Title	1	Identify the report as a scoping review.	1
		ABSTRACT	
Structured summary	2	Provide a structured summary that includes (as applicable): background, objectives, eligibility criteria, sources of evidence, charting methods, results, and conclusions that relate to the review questions and objectives.	1
		INTRODUCTION	
Rationale	3	Describe the rationale for the review in the context of what is already known. Explain why the review questions/objectives lend themselves to a scoping review approach.	2
Objectives	4	Provide an explicit statement of the questions and objectives being addressed with reference to their key elements (e.g., population or participants, concepts, and context) or other relevant key elements used to conceptualize the review questions and/or objectives.	2
		METHODS	
Protocol and registration	5	Indicate whether a review protocol exists; state if and where it can be accessed (e.g., a Web address); and if available, provide registration information, including the registration number.	N/A
Eligibility criteria	6	Specify characteristics of the sources of evidence used as eligibility criteria (e.g., years considered, language, and publication status), and provide a rationale.	2
Information sources*	7	Describe all information sources in the search (e.g., databases with dates of coverage and contact with authors to identify additional sources), as well as the date the most recent search was executed.	2
Search	8	Present the full electronic search strategy for at least 1 database, including any limits used, such that it could be repeated.	Supplementary Materials S1
Selection of sources of evidence†	9	State the process for selecting sources of evidence (i.e., screening and eligibility) included in the scoping review.	2
Data charting process‡	10	Describe the methods of charting data from the included sources of evidence (e.g., calibrated forms or forms that have been tested by the team before their use, and whether data charting was done independently or in duplicate) and any processes for obtaining and confirming data from investigators.	2
Data items	11	List and define all variables for which data were sought and any assumptions and simplifications made.	2
Critical appraisal of individual sources of evidence§	12	If done, provide a rationale for conducting a critical appraisal of included sources of evidence; describe the methods used and how this information was used in any data synthesis (if appropriate).	N/A
Synthesis of results	13	Describe the methods of handling and summarizing the data that were charted.	3
		RESULTS	
Selection of sources of evidence	14	Give numbers of sources of evidence screened, assessed for eligibility, and included in the review, with reasons for exclusions at each stage, ideally using a flow diagram.	Page 3, Figure 1
Characteristics of sources of evidence	15	For each source of evidence, present characteristics for which data were charted and provide the citations.	3–4, S2. Studies Included, S4. Data Abstraction
Critical appraisal within sources of evidence	16	If done, present data on critical appraisal of included sources of evidence (see item 12).	N/A

Results of individual sources of evidence	17	For each included source of evidence, present the relevant data that were charted that relate to the review questions and objectives.	S4. Data Abstraction Table
Synthesis of results	18	Summarize and/or present the charting results as they relate to the review questions and objectives.	3–4
DISCUSSION			
Summary of evidence	19	Summarize the main results (including an overview of concepts, themes, and types of evidence available), link to the review questions and objectives, and consider the relevance to key groups.	4
Limitations	20	Discuss the limitations of the scoping review process.	5
Conclusions	21	Provide a general interpretation of the results with respect to the review questions and objectives, as well as potential implications and/or next steps.	5
FUNDING			
Funding	22	Describe sources of funding for the included sources of evidence, as well as sources of funding for the scoping review. Describe the role of the funders of the scoping review.	N/A

JBI = Joanna Briggs Institute; PRISMA-ScR = Preferred Reporting Items for Systematic reviews and Meta-Analyses extension for Scoping Reviews. * Where *sources of evidence* (see second footnote) are compiled from, such as bibliographic databases, social media platforms, and Web sites. † A more inclusive/heterogeneous term used to account for the different types of evidence or data sources (e.g., quantitative and/or qualitative research, expert opinion, and policy documents) that may be eligible in a scoping review as opposed to only studies. This is not to be confused with *information sources* (see first footnote). ‡ The frameworks by Arksey and O'Malley (6) and Levac and colleagues (7) and the JBI guidance (4, 5) refer to the process of data extraction in a scoping review as data charting. § The process of systematically examining research evidence to assess its validity, results, and relevance before using it to inform a decision. This term is used for items 12 and 19 instead of "risk of bias" (which is more applicable to systematic reviews of interventions) to include and acknowledge the various sources of evidence that may be used in a scoping review (e.g., quantitative and/or qualitative research, expert opinion, and policy document). Abbreviation: N/A, not applicable.
